# Editorial Note: Ultra-compact quintuple-band terahertz metamaterial biosensor for enhanced blood cancer diagnostics

**DOI:** 10.1371/journal.pone.0349841

**Published:** 2026-05-21

**Authors:** The PLOS One Editors

The *PLOS One* Editors issue this Editorial Note to inform readers of the following issues that were noted after this article’s [1] publication:

- Compliance with PLOS Authorship Policy.
- Similarity with a study previously reported in [2] that was not cited or discussed in [1] and has one shared author.
- Contrary to the declaration in the Data Availability statement, the individual-level underlying data supporting the article’s results were not provided with the article.

The corresponding author acknowledged that the similar previous study [2] should have been cited and discussed in this article [1]. They stated that although both articles address terahertz metamaterial biosensing for blood cancer diagnostics, they represent distinct scientific contributions based on different design objectives and solutions. The Editors are satisfied that the two studies represent distinct contributions.

The individual-level underlying data supporting the article’s results are provided with this notice as Supporting Information files.

With this Editorial Note, the Editors consider the similarity and data availability concerns to be resolved.

## Supporting information

S1 File The six simulation models used in the study. These include the basic design models used in the development process, as well as the sixth model corresponding to the final proposed biosensor structure presented in the manuscript, in .CST file format.
(RAR)

S2 File Analyzed data used in the study. The dataset includes a total of 25 files covering all relevant simulation results and parameters necessary to support and replicate the findings of the manuscript, in .TXT file format.
(RAR)
